# Supplementary material for: Mechanical stretch induces hair regeneration through the alternative activation of macrophages
Source: Nat Commun. 2019 Apr 3;10:1524. doi: 10.1038/s41467-019-09402-8 (PMC6447615; doi:10.1038/s41467-019-09402-8)
Supplement: Supplementary file 1 — Supplementary Information [file 41467_2019_9402_MOESM1_ESM.pdf]

Mechanical stretch induces hair regeneration through the alternative activation of macrophages

Chu et al.

Supplementary Figures and Legends

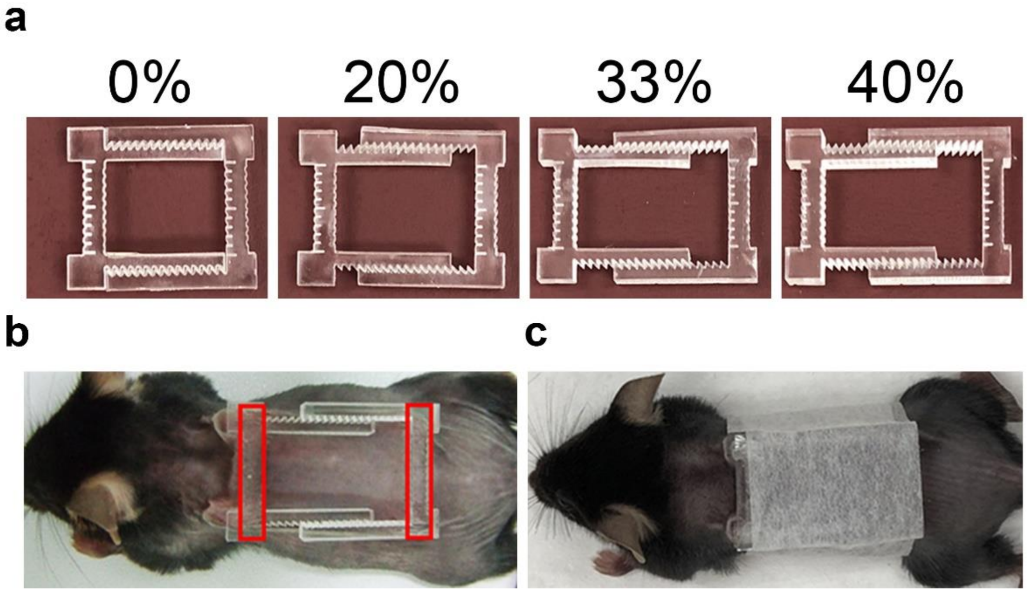

Supplementary Figure 1. In vivo stretching device and the procedures.
a The in vivo skin-stretching device was designed to create 0%, 20%, 33% and 40% strain. **b** The device can be fixed on mice skin by glue. The red area represents glue applying site. **c** Mice were taped around to prevent the detachment of the device.

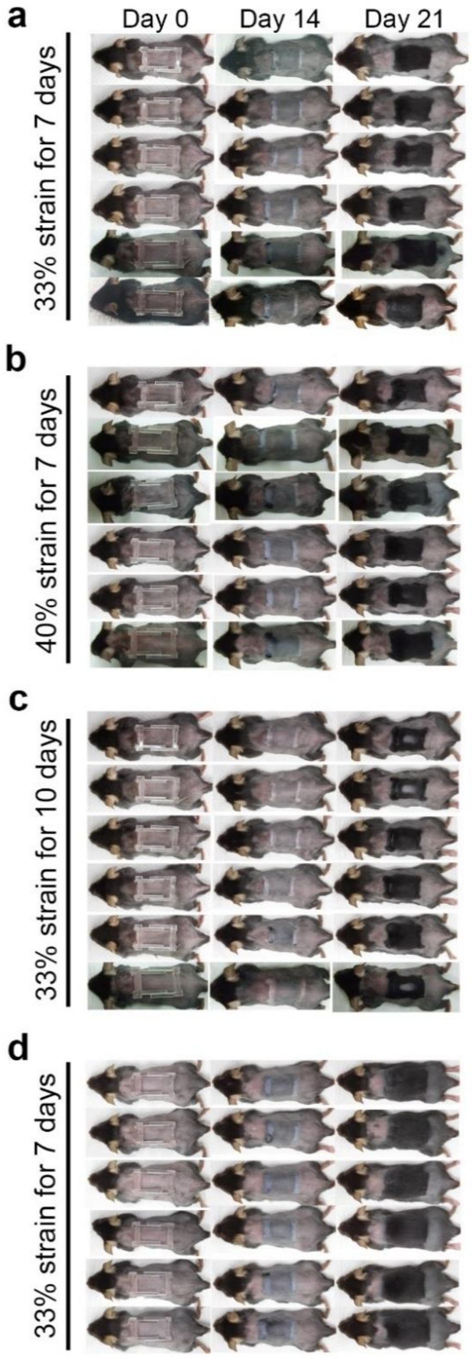

Supplementary Figure 2. Mechanical stretch induces hair regeneration in response to different strain and duration during the physiologic telogen and synchronized telogen phase.

a-c Hair regeneration occurred in response to 33% strain for 7-day duration (**a**), 40% strain for 7-day duration (**b**), and 33% strain for 10-day duration (**c**) during the physiologic telogen phase. **d** Hair regeneration occurred in response to 33% strain for 7-day duration during the synchronized telogen phase. Day 0 represents the day when strain was given; n = 6 for each group.

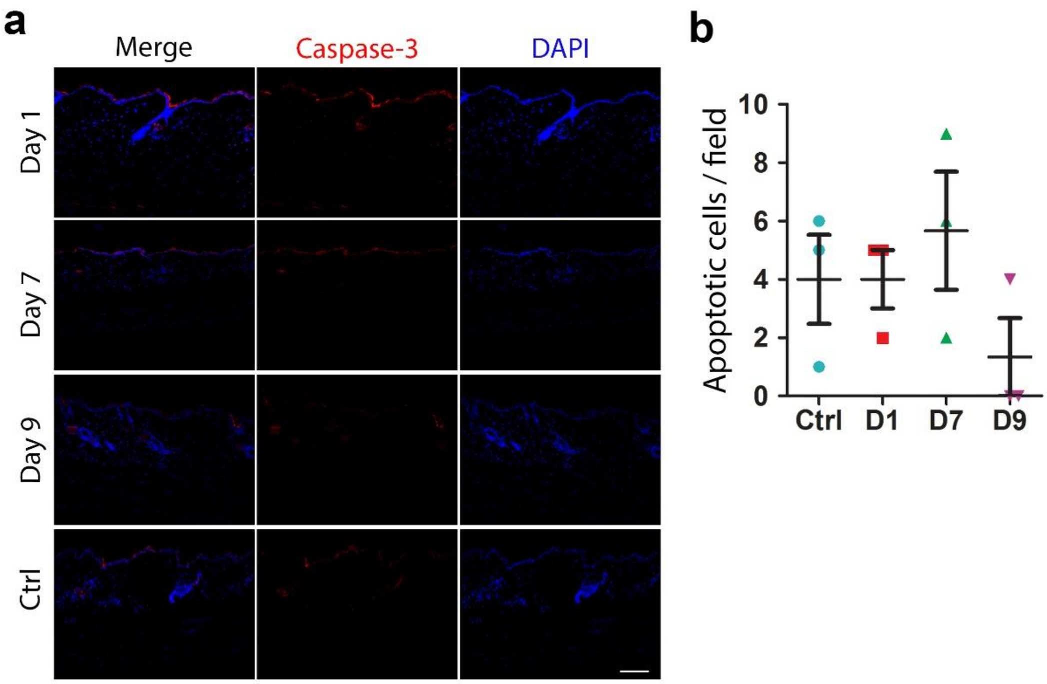

Supplementary Figure 3. No apparent apoptosis occurred in response to stretch. **a** Immunostaining for caspase-3 revealed only scarce apoptotic cells in the stretched skin. Scale bar = 100µm. **b** Quantification of apoptotic cells in response to stretch (day 1 and day 7) and stretch released (day 9). Apoptotic cells were counted in 200µm x 200µm field; n = 3 for each group. Data are presented as means ± SEM. Statistical significance was determined using ANOVA followed by a Bonferroni post hoc test.

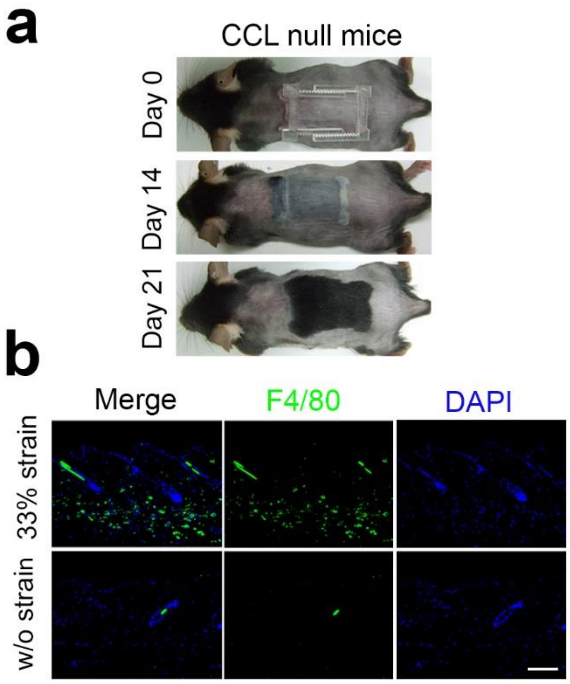

Supplementary Figure 4. Stretch-induced hair regeneration in CCL2 null mice.
a Stretch-induced hair regeneration failed to be abolished in CCL2-null mice; n = 3. **b** Immunofluorescence of back skin sections of CCL2 null mice revealed extensive F4/80$^+$ macrophage infiltration in response to stretch. Scale bar = 100μm.

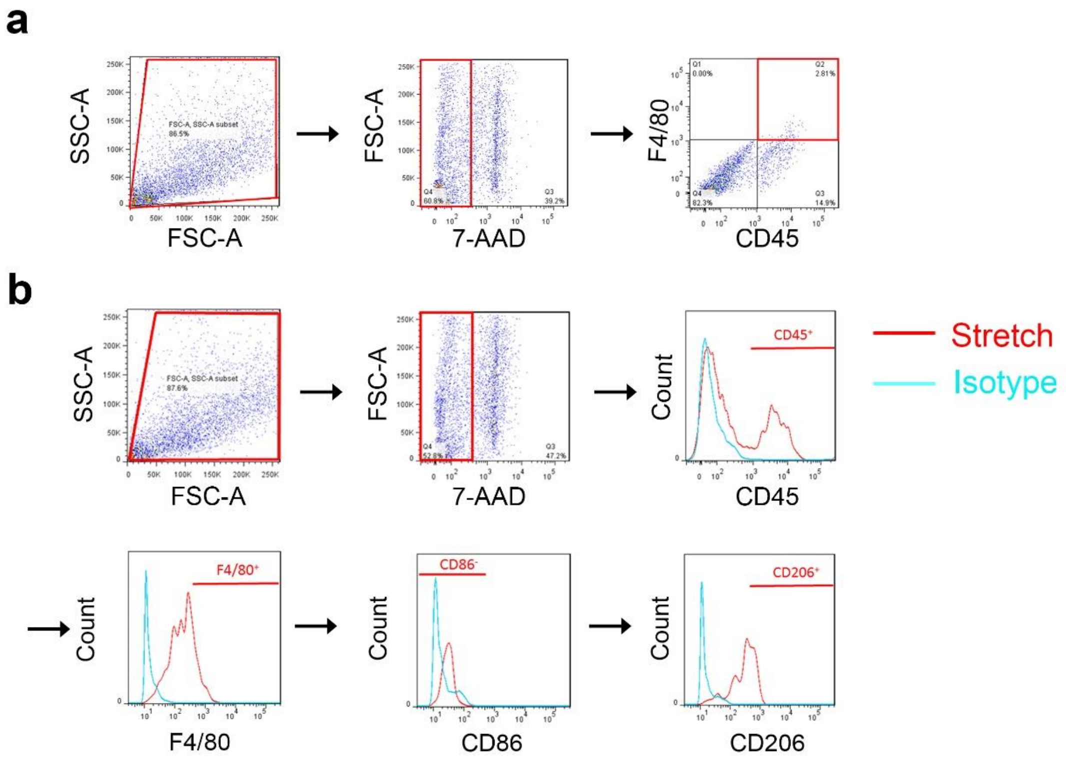

Supplementary Figure 5. Gating strategies used for flow cytometry.

a Gating Strategy for macrophages ($CD45^{+}F4/80^{+}$) presented on Fig. 4b. The representative gating strategy of flow cytometry (day 9) was shown. To identify macrophages, the dead cells were first excluded by 7-AAD staining, followed by positive staining of CD45 and F4/80. **b** Gating Strategy for M2 macrophages ($CD45^{+}F4/80^{+}CD86^{-}CD206^{+}$) presented on Fig. 5a. The representative gating strategy of flow cytometry (day 9) was shown. To identify M2 macrophages, the dead cells were first excluded by 7-AAD staining, followed by positive staining of CD45, F4/80 and CD206 as well as negative staining of CD86.

Supplementary Tables

Supplementary Table 1. Summary of top-ranked GO term enrichment of up-regulated genes in response to stretch.

Term	Count	%	P value	Benjamini
GO:0006955~immune response	62	15.12195	4.84E-31	7.44E-28
GO:0006952~defense response	52	12.68293	2.65E-23	2.03E-20
GO:0009611~response to wounding	42	10.2439	2.01E-19	1.03E-16
GO:0006954~inflammatory response	34	8.292683	1.16E-18	4.44E-16
GO:0042330~taxis	25	6.097561	4.67E-18	1.43E-15
GO:0006935~chemotaxis	25	6.097561	4.67E-18	1.43E-15
GO:0002252~immune effector process	22	5.365854	2.28E-13	5.85E-11
GO:0002684~positive regulation of immune system process	26	6.341463	1.90E-12	4.17E-10
GO:0002443~leukocyte mediated immunity	18	4.390244	4.72E-12	9.05E-10
GO:0007626~locomotory behavior	27	6.585366	8.52E-12	1.45E-09
GO:0002449~lymphocyte mediated immunity	16	3.902439	5.34E-11	8.20E-09
GO:0019724~B cell mediated immunity	15	3.658537	6.84E-11	9.55E-09
GO:0045087~innate immune response	18	4.390244	1.02E-10	1.31E-08
GO:0060326~cell chemotaxis	11	2.682927	1.03E-10	1.22E-08
GO:0030595~leukocyte chemotaxis	11	2.682927	1.03E-10	1.22E-08
GO:0002460~adaptive immune response based on somatic recombination of immune receptors built from immunoglobulin superfamily domains	16	3.902439	2.39E-10	2.62E-08
GO:0002250~adaptive immune response	16	3.902439	2.39E-10	2.62E-08
GO:0016064~immunoglobulin mediated immune response	14	3.414634	5.87E-10	6.02E-08
GO:0050900~leukocyte migration	12	2.926829	1.09E-09	1.04E-07

Supplementary Table 2. Summary of metabolic alteration in response to stretch revealed by GO term enrichment analysis.

Term	Count	%	P value
GO:0033559~unsaturated fatty acid metabolic process	7	1.70	1.02E-04
GO:0006631~fatty acid metabolic process	9	2.20	0.043863
GO:0006006~glucose metabolic process	10	4.18	4.01E-06
GO:0019318~hexose metabolic process	10	4.18	1.85E-05
mmu00010~glycolysis / gluconeogenesis	4	1.67	0.017801

Supplementary Table 3. Primer pairs used for quantitative RT-PCR.

Target gene	Primer	Sequence
Arginase1	forward	5’-gaatctgcatgggcaacc-3’
	reverse	5’-gaatcctggtacatctgggaac-3’
Bmp2	forward	5’-ccgtccctttcatttctcac-3’
	reverse	5’-ttggagaatgttccgtcgtt-3’
Ccl2	forward	5’-catccacgtgttggctca-3’
	reverse	5’-gatcatcttgctggtgaatgagt-3’
Ccl3	forward	5’-acctggaactgaatgcctga-3’
	reverse	5’-ctcaagcccctgctctacac-3’
Ccl6	forward	5’-ttatccttgtggctgtccttg-3’
	reverse	5’-tggagggttatagcgacgat-3’
Ccl7	forward	5’-ttctgtgcctgctgctcata-3’
	reverse	5’-ttgacatagcagcatgtggat-3’
Ccl12	forward	5’-tcctcaggtattggctggac-3’
	reverse	5’-gggacactggctgcttgt-3’
Ccl22	forward	5’-tcttgctgtggcaattcaga-3’
	reverse	5’-gagggtgacggatgtagtcc-3’
Fgf2	forward	5’-cggctctactgcaagaacg-3’
	reverse	5’-tgcttggagttgtagtttgacg-3’
Fgf10	forward	5’-cgggaccaagaatgaagact-3’
	reverse	5’-gcaacaactccgatttccac-3’
Hgf	forward	5’-cacccccttgggagtattgtg-3’
	reverse	5’-gggacatcagtctcattcacag-3’
Igf1	forward	5’-tcggcctcatagtacccact-3’
	reverse	5’-acgacatgatgtgtatctttattgc-3’

Il4	forward	5’-catcggcattttgaacgag-3’
	reverse	5’-cgagctcactctctgtggtg-3’
Kgf	forward	5’-tggctgacaccatgactagc-3’
	reverse	5’-ggctacaggctgtcgttttt-3’
Lef1	forward	5’-ctttggttaacgagtccgaaa-3’
	reverse	5’-ggcttgtctgaccacctca-3’
Vegf	forward	5’-gcagcttgagttaaacgaacg-3’
	reverse	5’-ggttcccgaaaccctgag-3’
Wnt7b	forward	5’-gcgtcctctacgtgaagctc-3’
	reverse	5’-tcttgttgcagatgatgttgg-3’
Wnt10a	forward	5’-tcctgttcttcctactgctgct-3’
	reverse	5’-aggatgtcgttgggtgct-3’
Ym1	forward	5’-ggtctgaaagacaagaacactgag-3’
	reverse	5’-gagaccatggcactgaacg-3’
